# Supplementary material for: Moving beyond MARCO
Source: PLoS One. 2023 Mar 24;18(3):e0283124. doi: 10.1371/journal.pone.0283124 (PMC10038243; doi:10.1371/journal.pone.0283124)
Supplement: S1 Appendix — Complete breakdown of hyper-parameters used to train models. (PDF) [file pone.0283124.s001.pdf]

Training Procedure

Unless otherwise specified all models were trained with the optimization hyper-parameters described in table S1. Data augmentation was also used to assist in regularization of the models, the particular data augmentation transformations that were used are listed in table S2. Model weights were initialised from using models from [1] which are pretrained upon the ImageNet dataset [2].

Table S1. Optimization hyper-parameters used during training.

**Parameter**	**Value**
Optimizer	AdamW [3]
Weight Decay	0.01
Learning Rate	0.0004
Learning Rate Schedule	None
Epochs	30
Batch Size	512
EMA Decay	0.9998
Label Smoothing	0.9

Table S2. Data augmentation transformations used during training.

**Transformation**
Horizontal Flip ($p = 0.5$)
Vertical Flip ($p = 0.5$)
Random Resized Crop (Scale $[0.08, 1]$, Ratio $[0.75, 1.33]$)
Color Jitter (factor 0.4)

References

1. Wightman R, Touvron H, Jégou H. ResNet strikes back: An improved training procedure in timm; 2021. Available from: `https://arxiv.org/abs/2110.00476`.

2. Deng J, Dong W, Socher R, Li LJ, Li K, Fei-Fei L. Imagenet: A large-scale hierarchical image database. In: 2009 IEEE conference on computer vision and pattern recognition. Ieee; 2009. p. 248–255.

3. Loshchilov I, Hutter F. Decoupled Weight Decay Regularization. In: International Conference on Learning Representations; 2019.Available from: `https://openreview.net/forum?id=Bkg6RiCqY7`.
