# Supplementary material for: Moving beyond MARCO
Source: PLoS One. 2023 Mar 24;18(3):e0283124. doi: 10.1371/journal.pone.0283124 (PMC10038243; doi:10.1371/journal.pone.0283124)
Supplement: S2 Appendix — S3 and S4 Tables show the correlation between labels after either one of two cycles of re-labelling. (PDF) [file pone.0283124.s002.pdf]

C3 dataset labelling

Tables S3, S4 show the correlations between the labels after either one or two cycles of re-labelling

Table S3. Confusion Matrix between human scores on the C3 dataset after the first round of re-labelling

	**Clear**	**Precipitate**	**Crystal**	**Other**
**Clear**	1685	383	55	466
**Precipitate**	-	2571	368	761
**Crystal**	-	-	2347	237
**Other**	-	-	-	407

https://www.overleaf.com/project/60876885763781e24a2f838f

The distribution of all pairs of scores by experts for images in the C3 dataset. Each image has been labelled at least twice.

Table S4. Confusion Matrix between human scores on the C3 dataset after the second round of re-labelling

	**Clear**	**Precipitate**	**Crystal**	**Other**
**Clear**	2089	854	163	944
**Precipitate**	-	3551	825	1556
**Crystal**	-	-	2544	516
**Other**	-	-	-	722

The distribution of all pairs of scores by experts for images in the C3 dataset. 'Hard' - *i.e.* ambiguous - images from the first round have been labelled again.
