# Supplementary material for: Moving beyond MARCO
Source: PLoS One. 2023 Mar 24;18(3):e0283124. doi: 10.1371/journal.pone.0283124 (PMC10038243; doi:10.1371/journal.pone.0283124)
Supplement: S3 Appendix — S1, S2 Figs show similar data to Figs 6 and 7 respectively, further decomposed into each groundtruth label. (PDF) [file pone.0283124.s003.pdf]

1 Projections

Figures S1 and S2 show the breakdown of classifications for the UMAP projections of either the MARCO classified or SimCLR analyzed MARCO validation set.

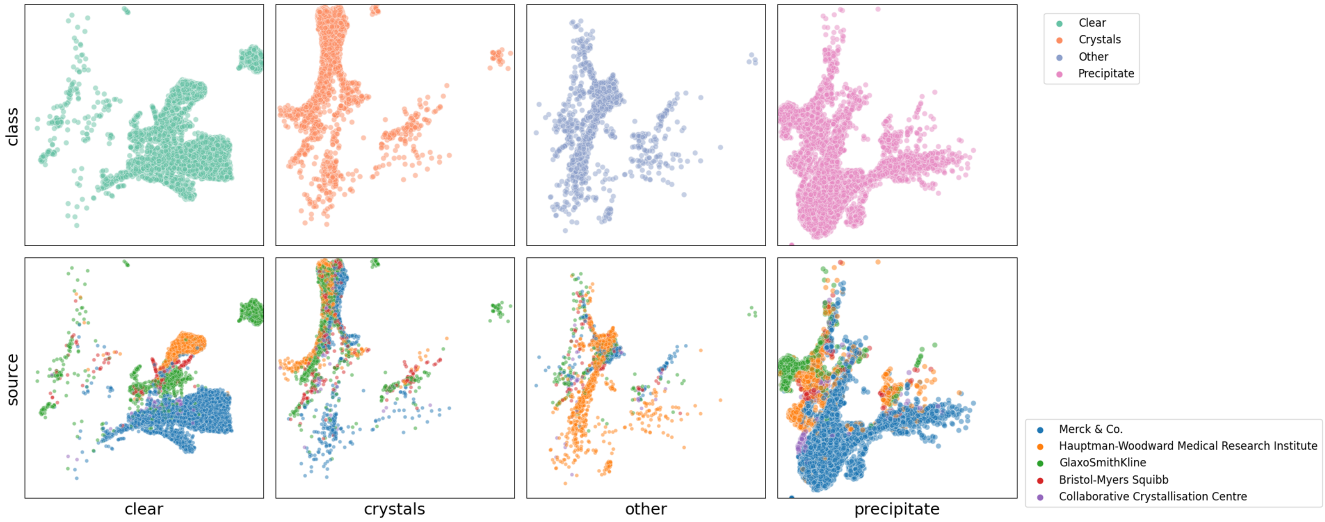

Fig S1. Breakdown by class, source and groundtruth class for the MARCO labelled MARCO validation data. A UMAP projection of the MARCO validation data, from the MARCO Inception V3 model. Decomposed into class, source, and groundtruth label (shown on the horizonal axis). The class labels are spread evenly throughout the projection, whereas the source attribute clusters in these projections.

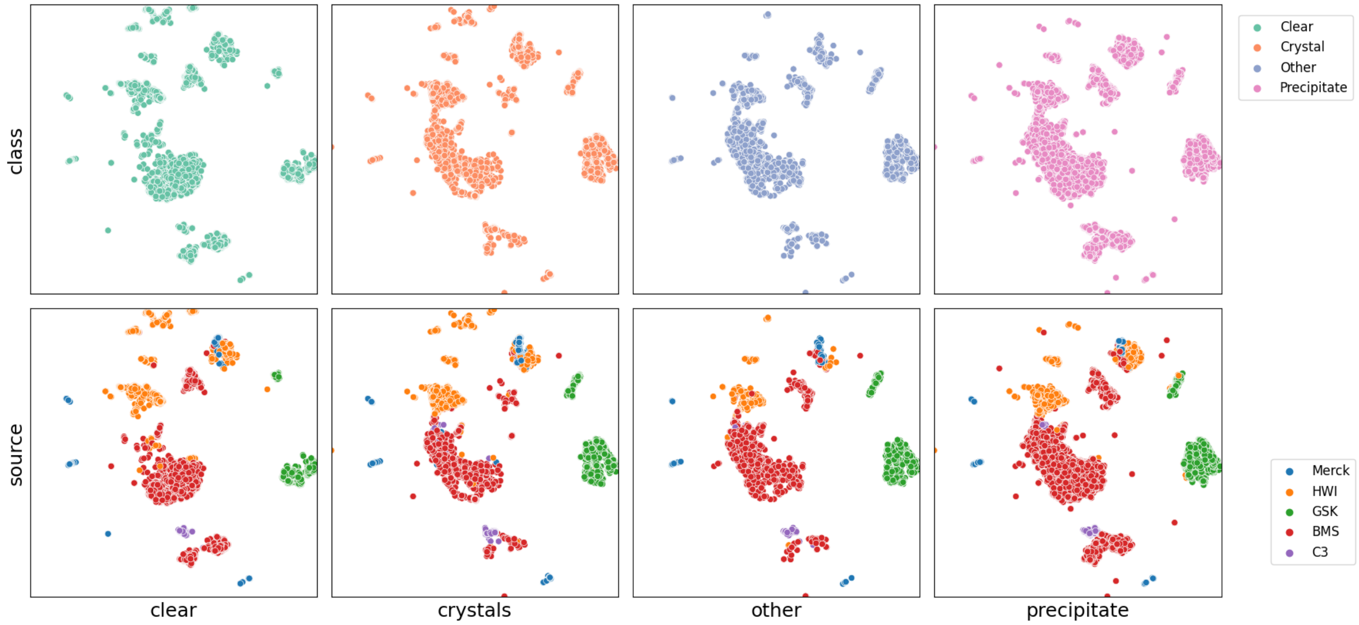

Fig S2. Breakdown by class, source and groundtruth class for the SimCLR (unsupervised) analysis of the MARCO validation data. A UMAP projection from the MARCO Inception V3 model. Decomposed into class, source, and groundtruth label (shown on the horizonal axis). The class labels are spread evenly throughout the projection, whereas the source attribute clusters in these projections.
