# Supplementary material for: Moving beyond MARCO
Source: PLoS One. 2023 Mar 24;18(3):e0283124. doi: 10.1371/journal.pone.0283124 (PMC10038243; doi:10.1371/journal.pone.0283124)
Supplement: Attachment — Submitted filename: response to reviewers.docx [file pone.0283124.s004.docx]

We thank the editor and the reviewers for their time and care in reviewing our manuscript. Please find our responses to the journal and the reviewer’s comments in black text below

1. Please ensure that your manuscript meets PLOS ONE's style requirements, including those for file naming. The PLOS ONE style templates can be found at 
<https://journals.plos.org/plosone/s/file?id=wjVg/PLOSOne_formatting_sample_main_body.pdf>

and 
<https://journals.plos.org/plosone/s/file?id=ba62/PLOSOne_formatting_sample_title_authors_affiliations.pdf>

2. Please review your reference list to ensure that it is complete and correct. If you have cited papers that have been retracted, please include the rationale for doing so in the manuscript text, or remove these references and replace them with relevant current references. Any changes to the reference list should be mentioned in the rebuttal letter that accompanies your revised manuscript. If you need to cite a retracted article, indicate the article’s retracted status in the References list and also include a citation and full reference for the retraction notice.

We have made the following changes in order to conform to the PONE standards.

- added corresponding author line to the authorship
- changed the current address symbol from dagger -> # [source](https://storage.googleapis.com/plos-published-prod/ba62/PLOSOne_formatting_sample_title_authors_affiliations.pdf?X-Goog-Algorithm=GOOG4-RSA-SHA256&X-Goog-Credential=wombat-sa%40plos-prod.iam.gserviceaccount.com%2F20221216%2Fauto%2Fstorage%2Fgoog4_request&X-Goog-Date=20221216T024947Z&X-Goog-Expires=86400&X-Goog-SignedHeaders=host&X-Goog-Signature=73cb2d71df08ceb48cb5da316e3031262e1bc06780d5136e46205fdf326848e04335261492b6a876af5be6d579488178864554f818dc13687f7fe7b1636694f721991a6d6ce3f8ac9fc21a61efa9e07656eaba7323d28b0dd7756843c6e21b8f91e7843158d75d81a8312d64d912ee7b491385db50986ff9008f3b022f1ddd0204212cb4c7b5f46ee524d77578b604b6b07d2924a29533231b4b1ac6661c42b993956a3133bd6672a91a396ec0917e2d5402a183f0402a18aef7b0c95f4b68ecc1e68e9d19f199f1785761294868f5eac4c4d098cc2c6da09ede4dadcbf8834464c610e926f7596e039c4af45904c4c8bf60d370b01f2f5d3e6334ce51089b7f)
- column headings in tables are now bold
- horizontal line between all lines in tables (not sure if necessary)
- combined fig 7&8 figures into single images
- renamed figures to match the figure naming from [here](https://journals.plos.org/plosone/s/figures)
- forced latex to insert the figures and tables after the paragraph where they are first cited
- Used the "retraction watch" service (retractionwatch.com) to check the status of the papers cited in the reference list.

Reviewer #1: This thoughtful and careful analysis of ML for detecting crystallization sheds new light on the standard reference tool, MARCO, and offers a path forward toward future improvement. In short, this manuscript is quite interesting and should be published. A couple of small things should, however, be fixed.

1) p.11: a comment by the first author was left in the text (and unaddressed).

The architecture has been explicitly defined, the sentence re-written as:

By training a SimCLR model with a ResNet50 backbone, using the unsupervised contrastive learning approach, we produced the plots in Fig 8.

2) p. 18: the notion of hallucination of features should be better explained and/or referenced.

The section about hallucination has been re-worked, and hopefully is now clearer:

During training the model can use the ground truth information to learn to hallucinate (imagine) detailed features from the low resolution image. This learnt relationship still applies to the MARCO test set because there is the same data distribution in the test set as there is in the training set. However, because the data distribution is most likely different between the C3 test set and the MARCO data the learnt relationship between low resolution images and the detailed features in the corresponding high-resolution images is no longer valid.

Reviewer #2: This paper is a welcome follow-up to the original MARCO study, which raised as many practical questions as it provided answers. The paper is well-written, and provides new insights to how the original findings from the MARCO study can be refined to become of practical use for a specific lab.

A few comments to the authors that are not commentary on the quality of the study, but could help direct further research:

1- While the ResNet class of models is a step up and a modernization of the original Inception-v3 model, it is by now also slightly outdated for many use-cases: modern Transformer-based architectures such as ViT (https://paperswithcode.com/paper/scaling-vision-transformers) and CoCa (https://paperswithcode.com/paper/coca-contrastive-captioners-are-image-text) have generally demonstrated superior performance *particularly* in a transfer learning setting. In other words, the world of computer vision is increasingly moving towards fine-tuning these (massive) models as opposed to training domain-specific models and seeing vastly improved transfer performance. Whether this finding transfers to the MARCO setting would be very interesting to find out.

SWIN Transformer architecture was investigated, but proved to be no more effective than ResNEt and significantly more resource intensive, to the point that only the low-resolution images could be classified as the GPU memory requirements increased by a factor of 8 when the image width doubled. Not only did the memory requirements for the high resolution images preclude us from continuing with the SWIN approach, we believe that the resources required would also make this approach inaccessible for most crystallography groups.

2- The fact that adding more C3 data with noisy labels ends up damaging performance (l453) suggests an opportunity to treat the domain transfer as a semi-supervised problem (https://paperswithcode.com/sota/semi-supervised-image-classification-on-2) which would have the benefit of potentially enabling the use of much more unlabeled data. More generally, if a large pool of unlabeled data is available, training a self-supervised model using contrastive loss on it, and then fine-tuning it using labeled data is fast becoming the

Yes, we are interested in this approach as well

Nit: l280 is missing a model name.

The architecture has been explicitly defined, the sentence re-written as

By training a SimCLR model with a ResNet50 backbone, using the unsupervised contrastive learning approach, we produced the plots in Fig 8.

Reviewer #3: Rose N. et al reported a follow-up study to enhance the original MARCO model. The MARCO performs well with the labelled training set from the MARCO publication (Bruno AE, 2018), but it does not perform as accurately on local datasets, which restricts its use in the ‘real world’. The authors have conducted extensive experiments to identify training settings that could help MARCO perform better at a local environment using ML (machine learning). The proposed model with the most recent machine learning architectures improves the accuracy of crystallization drop analysis outside of the test datasets which were used for the 2018 MARCO paper. They also considered the previous limitations and performed a new evaluation of the datasets for the present study.

General questions/remarks:
(1) Using “local” set from the same lab (C3) as part of the test set to train MARCO may introduce bias, even when those sets have been labelled by different crystallographers.

Yes, absolutely. Firstly, the data from C3 which was in the MARCO training set was produced from imagers that were decommissioned in 2016, so those images should not be related to the more recent images in the C3_test data used in this study. Secondly, the point of including local data into a larger training set is to induce some bias (overfitting) into the model, so that the local images are better classified at the expense of images from other sources.

(2) The performance of the new model is marginally better, but concerns about the quality and the diversity of the original MARCO images remain. Will it be more relevant to produce new and more diverse and controlled MARCO training and test sets?

It would be fabulous to create a bigger and better training set for crystallisation image analysis, but given the effort required (many years) to get the MARCO data, it is unlikely that a new and improved (labelled) dataset will ever be created. Hopefully we will get good at learning to use the vast amounts of unlabelled data which should be much easier to obtain.

(3) I am aware that sorting crystallization outcome is complex, but the “other” meta-label is too broad in my opinion. Poorly dispensed drops that are useless and drops with phase separation or spherulites that could be promising initial conditions may fall into this group. Can they be distinguished using the new ML model?

With further research, one might be able to subcluster within the other group to refine the “other” category. This would be useful, as at the moment the “other” classification is just a dumping ground for everything that isn’t a precipitate, crystal or clear.

(4) Do the imager settings influence the success of MARCO training with local sets?

Almost certainly imager settings are important, as otherwise all images from the Formulatrix imagers in the original MARCO dataset would be indistinguishable. It is likely that it is not only user-definable settings (e.g. FOV) that vary between the imagers in the original dataset, but also settings that are hardware linked (e.g., the age of the light source and the cleanliness of the camera lens). How much the imager settings influence the success of local implementations is hard to guess. Settings that mean that the images are out of focus (which are unfortunately more common than one would hope/expect) will clearly have a negative impact on local training.

The C3 local data used in training (and in the test set) were produced from two imagers, but we did not see if there was a difference in the performance of the local model on images from the two systems.

(5) Can the labeling differences between the test set and the local set affect the training's effectiveness? Since they were carried out by many individuals, the results' grading may vary.

This phenomenon creates the noise with which we struggled. We found the better the labels, the more effective the training (which is flip side to having worse performance by including more noisy data)

(6) line 280: What is “XX” architecture?

The architecture has been explicitly defined, the sentence re-written as:

By training a SimCLR model with a ResNet50 backbone, using the unsupervised contrastive learning approach, we produced the plots in Fig 8.
